# Performance of new nonparametric Tukey modified exponentially weighted moving average—Moving average control chart

**DOI:** 10.1371/journal.pone.0275260

**Published:** 2022-09-29

**Authors:** Khanittha Talordphop, Saowanit Sukparungsee, Yupaporn Areepong

**Affiliations:** Department of Applied Statistics, Faculty of Applied Science, King Mongkut’s University of Technology North Bangkok, Bangkok, Thailand; Sapienza University of Rome, ITALY

## Abstract

Control charts are an amazing and essential statistical process control (SPC) instrument that is commonly used in monitoring systems to detect a specific defect in the procedure. The mixed Tukey modified exponentially weighted moving average - moving average control chart (MMEM-TCC) with motivation detection ability for fewer shifts in the process mean under symmetric and non-symmetric distributions is proposed in this paper. Average run length (ARL), standard deviation of run length (SDRL), and median run length (MRL) were used as efficiency criteria in the Monte Carlo simulation, and their efficiency was compared to existing control charts. Furthermore, the expected ARL (EARL) is a method for evaluating the performance of control charts beyond a specific range of shift sizes. The distinguishing feature of the proposed chart is that it performs efficiently in detecting small to moderate shifts. There are applications for PM 2.5 and PM 10 data that demonstrate the performance of the proposed chart.

## Introduction

Control charts are a fantastic and important instrument of statistical process control (SPC) that is frequently used in monitoring system to detect a certain defect in the procedure. Effective monitoring is a fundamental component for improving the manufacturing process. Authors have been working to create an effective management scheme since Shewhart [1]. Next, Roberts [2] developed the exponentially weighted moving average (EWMA) control chart. Khoo [3] introduced moving average (MA) control chart. Patel and Divecha [4] created the modified exponentially weighted moving average (MEWMA) control chart. Khan et al. [5] developed a generalized form of MEWMA. Numerous adjustments have been proposed by the researchers to improve the quality of the work products.

Mixed control charts were developed to enhance the sensitivity of monitoring schemes. In this regard, Wong et al. [6] proposed a combined MA–Shewhart scheme for simple construction in the financial system. The mixed EWMA-CUSUM charts for process monitoring were explored by Abbas et al. [7]. The mixed CUSUM-EWMA chart (MCE) presented by Zaman et al. [8] is used to monitor the position of a process. Khan et al. [9] designed an EWMA control chart for exponential distributed quality based on moving average statistics. Taboran et al. [10] proposed the MA-EWMA charts while Sukparungsee et al. [11] reversed the MA-EWMA and suggested a mixed EWMA-MA charts. Anwar et al. [12] proposed using auxiliary data to create a modified-mxEWMA chart for improved system monitoring. Abbas et al. [13] created a new mixed EWMA–progressive mean (MEP) chart. Anwar et al. [14, 15] presented mixed MxMEC and MxMCE control charts for efficient process monitoring based on auxiliary data. Abid et al. [16, 17] presented a mixed homogeneously weighted moving average and cumulative sum (HWMA-CUSUM) and CUSUM-HWMA. Riaz et al. [18] created the progressive exponentially weighted moving average (PEWMA) chart. Saengsura et al. [19] created a mixed MA-CUSUM control chart for monitoring parameter change. The mixed MEWMA-MA (MMEM) and MA-MEWMA (MMME) charts for process monitoring presented by Talordphop et al. [20]. All of the foregoing mixed control charts performed well under normal conditions. If the process fails to meet the normality assumptions, the performance of the charts is challenged.

Nonparametric control charts were recommended by some researchers who proposed the control chart with distribution-free statistics. Nonparametric control charts are still a best alternative for monitoring the process because they are more resistant to outliers and have an extremely efficient quality in terms of shift detection when compared to their parametric counterparts in skewed and heavy-tailed procedures [21]. Tukey’s control chart has been widely used for individual processes since it was proposed by Alemi [22]. There are numerous benefits to using Tukey’s control chart, including its convenience of use in non-normal observations and when the process distribution is unknown, as well as appropriate control limit setup. Tukey’s control chart is also unaffected by unusual data, such as an outlier. Furthermore, many authors created and designed nonparametric control charts and mixed nonparametric control charts for a variety of situations, such as Sukparungsee [23] presented the robustness of asymmetric Tukey’s control charts in skew and non-skew populations. Khaliq et al. [24] created the EWMA-TCC. Riaz et al. [25] designed mixed Tukey EWMA-CUSUM chart (MEC-TCC). Taboran et al. [26, 27] investigated a Tukey MA-EWMA control chart and a Tukey MA-DEWMA control chart for the monitor mean process, respectively. Khaliq et al. [28] suggested a median-based design for Tukey and Tukey-EWMA control charts under subgrouping, and etc.

In this article, for fewer shifts in the mean procedures, the mixed Tukey MEWMA – MA (MMEM-TCC) control chart with confidence detection capability is proposed under symmetric and non-symmetric distributions. In Monte Carlo simulation, average run length (ARL), standard deviation of run length (SDRL), and median run length (MRL) are often used as performance indicators, and their quality was compared to appropriate control charts. Moreover, the expected ARL (EARL) is an alternative used to assess the performance of control charts beyond a specific range of shift sizes. Besides that, it was applied to two sets of potential environmental data.

## Design concepts of existing and proposed charts

This section describes the design concepts of parametric MA, MEWMA, MMME, MMEM and nonparametric TCC, MMME-TCC, and the proposed MMEM-TCC charts.

### Moving Average (MA) control chart

Assume that observations *X*_*t*_, for *t* = *1*,*2*,… are random sample from normal distribution with mean *μ*_0_ and variance *σ*^*2*^. The MA statistic at time *t* are calculated from the moving average at each period (*w*) as follows:

$$MA_{t}=\{\begin{array}{l} \frac{X_{t}+X_{t-1}+X_{t-2}+\ldots }{t},\;t<w\\ \frac{X_{t}+X_{t-1}+\ldots +X_{t-w+1}}{w},\;t\geq w. \end{array}$$ (1)

The mean and variance of MA statistic are given by:

$$E(X_{t})=E(MA_{t})=\mu_{0}$$ (2)

$$V(MA_{t})=\{\begin{array}{l} \frac{\sigma^{2}}{t},\;t<w\\ \frac{\sigma^{2}}{w},\;t\geq w. \end{array}$$ (3)

The upper (UCL) and lower control limit (LCL) of the MA chart can be constructed as:

$$UCL/LCL=\{\begin{array}{l} \mu_{0}\pm \frac{C_{1}\sigma }{\sqrt{t}},\;t<w\\ \mu_{0}\pm \frac{C_{1}\sigma }{\sqrt{w}},\;t\geq w \end{array}$$ (4)

where *μ*_0_ is the mean under process, *C*_*1*_ is the MA control chart’s control limits coefficient and *σ* is standard deviation of the process.

### Modified Exponentially Weighted Moving Average (MEWMA) **control chart**

Khan et al. [5] developed the structure of the MEWMA control chart. It is extremely effective in detecting both minor and major changes in the process. The statistic of MEWMA control chart that observation *X*_*t*_ for *t* = *1*,*2*,*…* from normal distribution is

$$M_{t}=\lambda X_{t}+(1-\lambda )M_{t-1}+k(X_{t}-X_{t-1}),t=1,2,\ldots ,$$ (5)

where *λ* is a smoothing parameter, *0*≤*λ*≤*1* and *k* is an additional parameter (*k*≠*0*). The additional parameter *k* is often used to boost the effectiveness of traditional charts in detecting shifts that they are not optimally designed to detect. Then the mean and the asymptotic variance when *t*→∞ of *M*_*t*_ are:

$$E(M_{t})=\mu_{0}$$ (6)

$$V(M_{t})=\sigma^{2}[\frac{\left(\lambda +2\lambda k+2k^{2}\right)}{2-\lambda }].$$ (7)

The time-varying control boundaries of MEWMA chart are given by

$$UCL/LCL=\mu_{0}\pm C_{2}\sigma \sqrt{\frac{\left(\lambda +2\lambda k+2k^{2}\right)}{\left(2-\lambda \right)}}$$ (8)

where *C*_*2*_ is the control limits coefficient for the MEWMA control chart. *μ*_*0*_ and *σ*^*2*^ are the mean and variance of the process, respectively.

### Mixed MA - MEWMA (MMME) control chart

The parametric control charts MA and MEWMA were combined to create this chart. The *M*_*t*_ statistic of the MEWMA chart should be used as an input to the MA chart (Eq 1). The MMME statistic control chart can be developed as

$$MA_{t}=\{\begin{array}{l} \frac{M_{t}+M_{t-1}+M_{t-2}+\ldots }{t},\;t<w\\ \frac{M_{t}+M_{t-1}+\ldots +M_{t-w+1}}{w},\;t\geq w. \end{array}$$ (9)

The asymptotical upper and lower control limits of the MMME chart are given as follow:

$$UCL/LCL=\{\begin{array}{l} \mu_{M}\pm C_{3}\sqrt{\left(\frac{\sigma_{M}^{2}}{t})(\frac{\lambda +2\lambda k+2k^{2}}{2-\lambda }\right)},\;t<w\\ \mu_{M}\pm C_{3}\sqrt{\left(\frac{\sigma_{M}^{2}}{w})(\frac{\lambda +2\lambda k+2k^{2}}{2-\lambda }\right)},\;t\geq w \end{array}$$ (10)

where *C*_*3*_ is the control limits coefficient for the MMME control chart. *μ*_*M*_ is the mean and $\sigma_{M}^{2}$ is variance of the MEWMA respectively.

### Mixed MEWMA - MA (MMEM) control chart

Similarly, the MMEM chart was generated from combining the MEWMA and MA control chart. The statistic of MMEM control chart is defined as

$$M_{t}=\lambda MA_{t}+(1-\lambda )M_{t-1}+k(MA_{t}-MA_{t-1}),\;t=1,2,\ldots$$ (11)

where *M*_*t*_ is the MMEM statistic at time *i*^*th*^, *MA*_*t*_ is the MA statistic at time *i*^*th*^, *λ* is a smoothing parameter between 0 to 1 and *k* is a additional parameter (*k*≠0). Thus, the upper and lower control limits of the MMEM chart are given as follow:

$$UCL/LCL=\mu_{MA}\pm C_{4}\sqrt{\left(\frac{\sigma_{MA}^{2}}{w})(\frac{\lambda +2\lambda k+2k^{2}}{2-\lambda }\right)}$$ (12)

where *C*_*4*_ is the control limits coefficient for the MMEM control chart. *μ*_*MA*_ is the mean and $\sigma_{MA}^{2}$ is variance of the MA respectively.

### Tukey’s control chart (TCC)

The TCC is the nonparametric control chart. The control limits are:

$$\begin{array}{l} UCL=Q_{3}+C(IQR)\\ LCL=Q_{1}-C(IQR) \end{array}$$ (13)

where IQR is the interquartile range (*Q*_3_−*Q*_1_), *Q*_*1*_ and *Q*_*3*_ are the first and the third quartiles and *C* is the control limits coefficient for the TCC.

### Mixed Tukey MA - MEWMA (MMME-TCC) control chart

The MMME-TCC control chart was designed by combining the MMME and TCC control chart, which uses the statistic of MMME. The upper and lower control limits of the MMME-TCC chart are given as follow: where *t < w*

$$\begin{array}{l} UCL=Q_{3}+C_{5}(IQR)\sqrt{\left(\frac{1}{t})(\frac{\lambda +2\lambda k+2k^{2}}{2-\lambda }\right)}\\ LCL=Q_{1}-C_{5}(IQR)\sqrt{\left(\frac{1}{t})(\frac{\lambda +2\lambda k+2k^{2}}{2-\lambda }\right)} \end{array}$$ (14)

where *t* ≥ *w*

$$\begin{array}{l} UCL=Q_{3}+C_{5}(IQR)\sqrt{\left(\frac{1}{w})(\frac{\lambda +2\lambda k+2k^{2}}{2-\lambda }\right)}\\ LCL=Q_{1}-C_{5}(IQR)\sqrt{\left(\frac{1}{w})(\frac{\lambda +2\lambda k+2k^{2}}{2-\lambda }\right)} \end{array}$$ (15)

where *C*_*5*_ is the control limits coefficient for the MMME-TCC control chart. *λ* is the weighing parameter of the data in the past, such that *0*<*λ*≤*1*. IQR is the inter quartile range, *Q*_*1*_ and *Q*_*3*_ are the first and third quartiles.

### Mixed Tukey MEWMA - MA (MMEM-TCC) control chart

Likewise, the MMEM-TCC control chart is a combination of the MMEM and TCC control charts, thus uses the statistic of MMEM. The upper and lower control limits of the MMEM-TCC chart can be developed as

$$\begin{array}{l} UCL=Q_{3}+C_{6}(IQR)\sqrt{\left(\frac{1}{w})(\frac{\lambda +2\lambda k+2k^{2}}{2-\lambda }\right)}\\ LCL=Q_{1}-C_{6}(IQR)\sqrt{\left(\frac{1}{w})(\frac{\lambda +2\lambda k+2k^{2}}{2-\lambda }\right)} \end{array}$$ (16)

where *C*_*6*_ is the control limits coefficient for the MMEM-TCC control chart. *λ* is the weighing parameter of the data in the past, such that *0*<*λ*≤*1*. IQR is the inter quartile range, *Q*_*1*_ and *Q*_*3*_ are the first and third quartiles.

## Performance evaluation

The statistical effectiveness of control charts is traditionally calculated in terms of the average run length (ARL) based on the mean of the run length distribution. In other words, ARL is referred to as the average number of observations mapped on a control chart before an out-of-control sensor or a false alarm happens. ARL_0_ happens when the operation is under control, whereas ARL_1_ appears when the operation is out of control. When the operation is on goal (the mean is at the desirable level), ARL_0_ should be large, but ARL_1_ should be small in order to detect a change in the process mean quickly [29]. To achieve the best run length profile results, the Monte Carlo technique is used to simulate the simulation results when the process is under control *ARL*_*0*_ = *370* with 200,000 replications and taking samples of size *(n)* 10,000. In this article, ARL, standard deviation of run length (SDRL), and median run length (MRL) are convenient measures for evaluating performance. The solution for run length is as follows:

$$ARL=\frac{\sum_{t=1}^{N}RL_{t}}{N}$$ (17)

$$SDRL=\sqrt{E{\left(RL\right)}^{2}-ARL^{2}}$$ (18)

MRL=Median(RL) (19)

where *RL*_*t*_ is the number of samples required before the process becomes uncontrollable for the first time in the simulation at round *t* and *N* is the number of experiment repetitions.

Moreover, the ARL can only be used to monitor the effectiveness of a scheme if the accurate shift size in the process is known, which is inconvenient in real life situations. The expected ARL (EARL) is an alternative used to assess the performance of control charts beyond a specific range of shift sizes [30, 31]. It is essential to consider the EARL when determining the overall range of shifts (*δ*_*1*_,*δ*_*2*_). The EARL is defined as follow:

$$EARL=\frac{1}{\delta_{2}-\delta_{1}}\int_{\delta_{1}}^{\delta_{2}}ARL(\delta )d\delta$$ (20)

where *δ*_*1*_ and *δ*_*2*_ represent the lower and upper bounds of the shift, respectively.

## Simulation results

The goal of this study was to examine the efficiency with which the proposed chart detected a change in the process mean with MA, MEWMA, MMME, MMEM, and MMME-TCC control charts for symmetric distributions: Normal(0,1), Laplace(0,1), and non-symmetric distributions: Exponential(1), Gamma(4,1). The control chart with the lowest ARL_1_, SDRL, and MRL was found to be the most efficient, as indicated by the bold value in the tables. Tables 1–4 show the simulation results for various combination of ARL_0_ = 370, w = 5, λ = 0.25, k = −0.125 and shift constant (between -4 and 4). It is demonstrated that the proposed chart displays smaller ARL_1_ values than existing charts.

**Table 1:** The run length attributes of the proposed MMEM-TCC chart and existing control charts for normal distribution.

shift	MA			MEWMA			MMME			MMEM			MMME-TCC			MMEM-TCC		
	*C*_*1*_ = *2*.*882*			*C*_*2*_ = *2*.*199*			*C*_*3*_ = *5*.*08*6			*C*_*4*_ = *5*.*118*			*C*_*5*_ = *16*.*610*			*C*_*6*_ = 5.*320*		
	ARL	SDRL	MRL	ARL	SD RL	M RL	ARL	SD RL	MRL	ARL	SDRL	MRL	ARL	SD RL	MRL	ARL	SDRL	MRL
-4.00	**1.00**	0.00	1	1.07	0.00	1	2.07	0.00	2	1.02	0.00	1	**1.00**	0.00	1	1.02	0.00	1
-3.00	1.10	0.00	1	1.49	0.00	1	2.70	0.00	3	1.24	0.00	1	**1.01**	0.00	1	1.24	0.00	1
-2.00	1.99	0.00	2	2.69	0.00	2	4.02	0.00	3	2.13	0.00	2	**1.32**	0.00	**1**	2.12	0.00	2
-1.50	3.75	0.01	3	4.28	0.01	4	5.39	0.00	5	3.36	0.00	3	**2.42**	0.01	**1**	3.33	0.00	3
-1.00	10.09	0.02	7	9.10	0.01	7	9.42	0.01	8	7.11	0.01	6	7.39	0.02	6	**7.03**	0.01	6
-0.75	20.60	0.04	15	16.41	0.03	13	15.66	0.03	12	12.96	0.03	10	16.03	0.04	10	**12.80**	0.03	10
-0.50	51.32	0.11	36	38.44	0.08	28	34.72	0.07	26	31.39	0.07	22	39.48	0.10	27	**30.71**	0.07	**22**
-0.25	162.62	0.36	113	129.24	0.28	90	117.59	0.25	83	113.74	0.25	78	135.75	0.34	92	**110.46**	0.25	**76**
-0.1	311.10	0.69	215	290.96	0.64	203	280.52	0.61	196	278.99	0.61	191	305.18	0.67	187	**263.95**	0.61	**181**
-0.05	350.63	0.78	243	347.39	0.77	242	343.25	0.76	239	342.89	0.76	233	359.37	0.78	220	**322.08**	0.75	220
0	370.70	0.83	257	370.67	0.82	258	370.26	0.82	257	370.11	0.82	253	370.04	0.85	252	370.25	0.86	252
0.05	353.30	0.79	244	346.66	0.77	240	342.64	0.75	239	340.73	0.75	233	350.47	0.85	237	**316.66**	0.73	**216**
0.1	309.36	0.69	213	289.90	0.64	201	279.66	0.61	195	278.39	0.61	190	290.14	0.76	201	**256.89**	0.59	**175**
0.25	161.35	0.36	112	129.30	0.28	91	117.89	0.25	83	113.93	0.25	78	128.33	0.41	91	**106.16**	0.24	**73**
0.50	51.48	0.11	36	38.37	0.08	28	34.66	0.07	26	31.42	0.07	22	37.96	0.12	28	**29.89**	0.07	**21**
0.75	20.53	0.04	15	16.38	0.03	13	15.68	0.03	12	12.99	0.03	10	16.28	0.05	12	**12.55**	0.02	**9**
1.00	10.05	0.02	7	9.05	0.01	7	9.41	0.01	8	7.11	0.01	6	8.28	0.21	5	**6.93**	0.01	5
1.50	3.75	0.01	3	4.29	0.01	4	5.39	0.00	5	3.36	0.00	3	**2.61**	0.01	**1**	3.29	0.00	3
2.00	1.99	0.00	2	2.69	0.00	2	4.01	0.00	4	2.13	0.00	2	**1.36**	0.00	**1**	2.10	0.00	2
3.00	1.10	0.00	1	1.49	0.00	1	2.70	0.00	3	1.24	0.00	1	**1.01**	0.00	1	1.23	0.00	1
4.00	**1.00**	0.00	1	1.07	0.00	1	2.07	0.00	2	1.02	0.00	1	**1.00**	0.00	1	1.01	0.00	1

**Table 2:** The run length attributes of the proposed MMEM-TCC chart and existing control charts for Laplace distribution.

shift	MA			MEWMA			MMME			MMEM			MMME-TCC			MMEM-TCC		
	*C*_*1*_ = *2*.*119*			*C*_*2*_ = *1*.*749*			*C*_*3*_ = *3*.*826*			*C*_*4*_ = *5*.*453*			*C*_*5*_ = *19*.*199*			*C*_*6*_ = *6*.*479*		
	ARL	SDRL	MRL	ARL	SD RL	M RL	ARL	SD RL	MRL	ARL	SDRL	MRL	ARL	SDRL	MRL	ARL	SDRL	MRL
-4.00	1.20	0.00	1	1.91	0.00	2	3.05	0.00	3	1.36	0.00	1	**1.03**	0.00	1	1.37	0.00	1
-3.00	1.98	0.00	2	2.88	0.00	3	4.02	0.00	4	2.12	0.00	2	**1.25**	0.00	**1**	2.12	0.00	2
-2.00	5.35	0.01	4	5.93	0.01	5	6.13	0.01	6	4.04	0.01	3	**3.36**	0.01	**1**	4.06	0.01	3
-1.50	12.54	0.03	9	11.28	0.02	9	9.41	0.01	8	7.17	0.01	6	**7.13**	0.02	6	9.07	0.01	6
-1.00	38.53	0.08	27	30.39	0.06	22	21.31	0.04	16	18.78	0.04	14	30.89	0.08	20	**18.72**	0.04	14
-0.75	74.04	0.16	52	58.44	0.12	42	39.99	0.08	29	37.09	0.08	26	60.94	0.15	40	**37.02**	0.08	26
-0.50	146.84	0.33	102	122.72	0.27	87	89.39	0.19	64	86.34	0.19	60	121.82	0.29	81	**86.24**	0.19	60
-0.25	276.69	0.62	192	255.95	0.57	178	219.27	0.48	154	217.43	0.50	148	226.34	0.54	152	**217.04**	0.50	148
-0.1	351.87	0.79	243	346.17	0.77	240	335.55	0.74	232	335.07	0.77	230	323.42	0.67	229	**322.54**	0.74	221
-0.05	364.12	0.81	252	363.77	0.81	252	362.03	0.79	250	362.02	0.83	248	341.99	0.69	235	**340.57**	0.78	233
0	370.75	0.83	257	370.56	0.82	257	370.55	0.82	258	370.54	0.85	254	370.56	0.84	255	370.74	0.86	252
0.05	364.68	0.82	253	364.27	0.81	253	361.58	0.79	251	360.68	0.83	247	328.58	0.67	189	**328.57**	0.76	189
0.1	350.53	0.79	243	346.95	0.77	241	335.94	0.74	235	333.97	0.77	229	301.52	0.64	180	**299.29**	0.69	180
0.25	275.59	0.62	191	255.68	0.57	179	218.33	0.48	153	217.37	0.49	149	207.27	0.49	139	**189.78**	0.44	129
0.50	146.61	0.33	102	122.73	0.27	86	89.22	0.19	64	86.29	0.19	60	109.58	0.26	73	**76.19**	0.17	53
0.75	74.33	0.16	52	58.39	0.12	42	40.06	0.08	29	37.26	0.08	26	54.25	0.13	36	**33.64**	0.07	24
1.00	38.59	0.08	27	30.42	0.06	22	21.31	0.04	16	18.76	0.04	14	27.79	0.07	18	**17.23**	0.03	13
1.50	12.57	0.03	9	11.29	0.02	9	9.41	0.01	8	7.17	0.01	6	**6.77**	0.02	5	8.29	0.01	5
2.00	5.34	0.01	4	5.95	0.01	5	6.14	0.01	6	4.05	0.01	3	**3.14**	0.01	**1**	3.86	0.01	3
3.00	1.98	0.00	2	2.88	0.00	3	4.01	0.00	4	2.11	0.00	2	**1.23**	0.00	**1**	2.05	0.00	2
4.00	1.19	0.00	1	1.92	0.00	2	3.05	0.00	3	1.37	0.00	1	**1.03**	0.00	1	1.32	0.00	1

Note: The bold represents the least number of *ARL*_*1*_, SDRL and MRL

**Table 3:** The run length attributes of the proposed MMEM-TCC chart and existing control charts for exponential distribution.

shift	MA			MEWMA			MMME			MMEM			MMME-TCC			MMEM-TCC		
	*C*_*1*_ = *3*.*338*			*C*_*2*_ = *2*.*691*			*C*_*3*_ = *5*.*617*			*C*_*4*_ = *6*.*064*			*C*_*5*_ = *20*.*794*			*C*_*6*_ = *7*.*174*		
	ARL	SDRL	MRL	ARL	SD RL	M RL	ARL	SD RL	MRL	ARL	SDRL	MRL	ARL	SDRL	MRL	ARL	SDRL	MRL
0	370.64	0.83	256	370.53	0.82	259	370.35	0.82	258	370.29	0.82	185	370.50	0.84	254	370.46	0.92	240
0.05	253.22	0.57	175	252.04	0.55	176	246.95	0.54	172	241.09	0.51	125	247.29	0.58	168	**213.04**	0.54	124
0.1	179.68	0.40	124	178.76	0.39	126	172.60	0.37	122	164.47	0.37	87	174.36	0.41	118	**148.55**	0.37	86
0.25	79.73	0.18	55	80.25	0.17	57	75.43	0.15	54	68.32	0.15	39	75.27	0.18	50	**64.59**	0.16	38
0.50	31.41	0.07	22	33.38	0.07	25	32.42	0.06	24	26.89	0.06	17	28.73	0.07	19	**26.44**	0.06	16
0.75	16.93	0.04	12	19.34	0.04	15	19.46	0.03	15	15.15	0.03	10	15.13	0.04	10	**15.10**	0.03	10
1.00	10.97	0.02	8	13.29	0.02	10	13.88	0.02	11	10.28	0.02	7	**9.47**	0.02	6	10.55	0.02	8
1.50	6.05	0.01	4	8.09	0.01	7	9.07	0.01	8	6.32	0.01	5	**5.10**	0.01	2	6.55	0.01	5
2.00	4.14	0.01	3	5.85	0.01	5	7.01	0.00	6	4.67	0.00	4	**3.42**	0.01	1	4.86	0.01	4
3.00	2.58	0.01	2	3.84	0.01	3	5.04	0.01	5	3.21	0.00	3	**2.15**	0.00	1	3.31	0.01	3
4.00	1.97	0.00	1	2.92	0.00	2	4.06	0.00	4	2.51	0.00	2	**1.67**	0.00	1	2.59	0.00	2

Note: The bold represents the least number of *ARL*_*1*_, SDRL and MRL

**Table 4:** The run length attributes of the proposed MMEM-TCC chart and existing control charts for gamma distribution.

shift	MA			MEWMA			MMME			MMEM			MMME-TCC			MMEM-TCC		
	*C*_*1*_ = *1*.*512*			*C*_*2*_ = *1*.*382*			*C*_*3*_ = *4*.*272*			*C*_*4*_ = *9*.*460*			*C*_*5*_ = *17*.*910*			*C*_*6*_ = *12*.*474*		
	ARL	SDRL	MRL	ARL	SD RL	M RL	ARL	SD RL	MRL	ARL	SDRL	MRL	ARL	SDRL	MRL	ARL	SDRL	MRL
0	370.35	0.83	258	370.51	0.83	257	370.27	0.83	257	370.15	0.82	186	370.45	0.82	256	370.31	0.82	256
0.05	284.03	0.64	197	281.59	0.63	197	282.94	0.63	196	253.05	0.62	141	253.56	0.62	141	**162.86**	0.60	137
0.1	220.39	0.49	153	219.15	0.49	152	219.06	0.49	152	204.56	0.47	101	204.87	0.48	101	**126.62**	0.42	**85**
0.25	109.48	0.24	76	109.95	0.24	75	108.55	0.24	75	96.54	0.22	59	103.87	0.22	70	**60.31**	0.21	**38**
0.50	41.71	0.09	29	44.61	0.09	28	41.51	0.09	28	35.56	0.08	20	40.43	0.08	27	**21.61**	0.07	**11**
0.75	19.61	0.04	14	19.52	0.04	14	19.44	0.04	13	15.24	0.02	12	16.09	0.03	12	**10.59**	0.03	**8**
1.00	10.75	0.02	8	10.65	0.01	8	9.44	0.02	8	8.14	0.01	7	**6.63**	0.02	**5**	6.81	0.02	6
1.50	4.52	0.01	3	4.50	0.01	4	4.50	0.01	4	4.47	0.00	4	**3.75**	0.01	3	4.39	0.01	3
2.00	2.52	0.00	2	2.86	0.00	3	2.72	0.00	2	3.23	0.00	3	**2.50**	0.00	2	3.78	0.00	3
3.00	1.32	0.00	1	1.92	0.00	2	1.33	0.00	1	2.12	0.00	2	**1.30**	0.00	1	3.29	0.00	2
4.00	1.06	0.00	1	1.39	0.00	1	1.07	0.00	1	1.57	0.00	2	**1.01**	0.00	1	2.88	0.00	2

Note: The bold represents the least number of *ARL*_*1*_, SDRL and MRL

**Fig 1:**
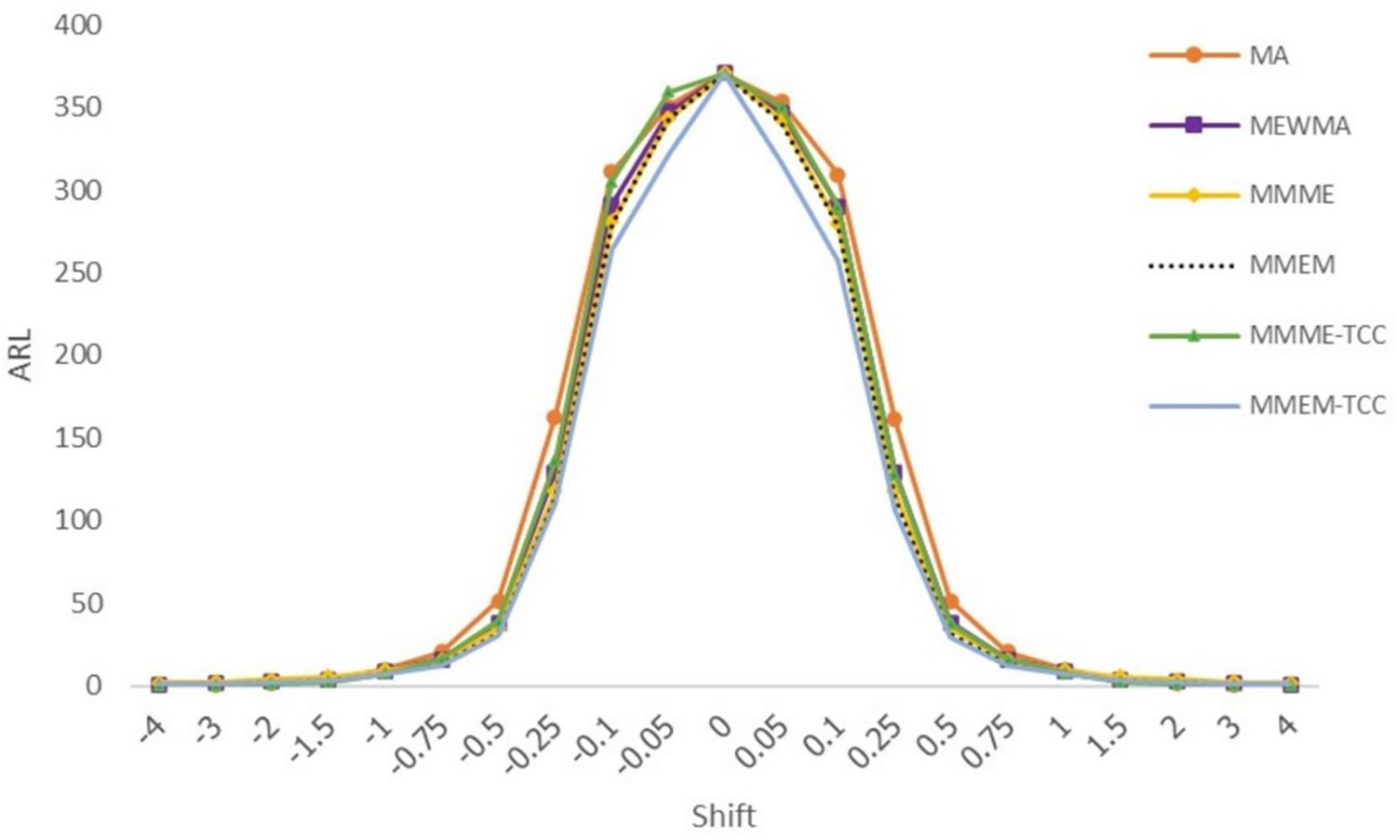
Displays of ARL curves of the MA, MEWMA, MMME, MMEM, MMME-TCC, and MMEM-TCC control charts for normal distribution.

On observing Table 1 and Fig 1 when the process follows normal distribution, at shifts ±0.05, ±0.10, ±0.25, ±0.50, ±0.75, ±1.00, the proposed chart at *C*_*6*_ = *5*.*320* outperforms other charts in terms of detection whereas the MMME-TCC control chart is the most useful in detecting changes when shifts ±1.50, ±2.00, ±3.00. Moreover, when detecting shift at ±4.00, the MMME-TCC and the MA control chart outperform. Considering the SDRL and MRL values, it was found that the results were consistent with ARL_1_.

**Fig 2:**
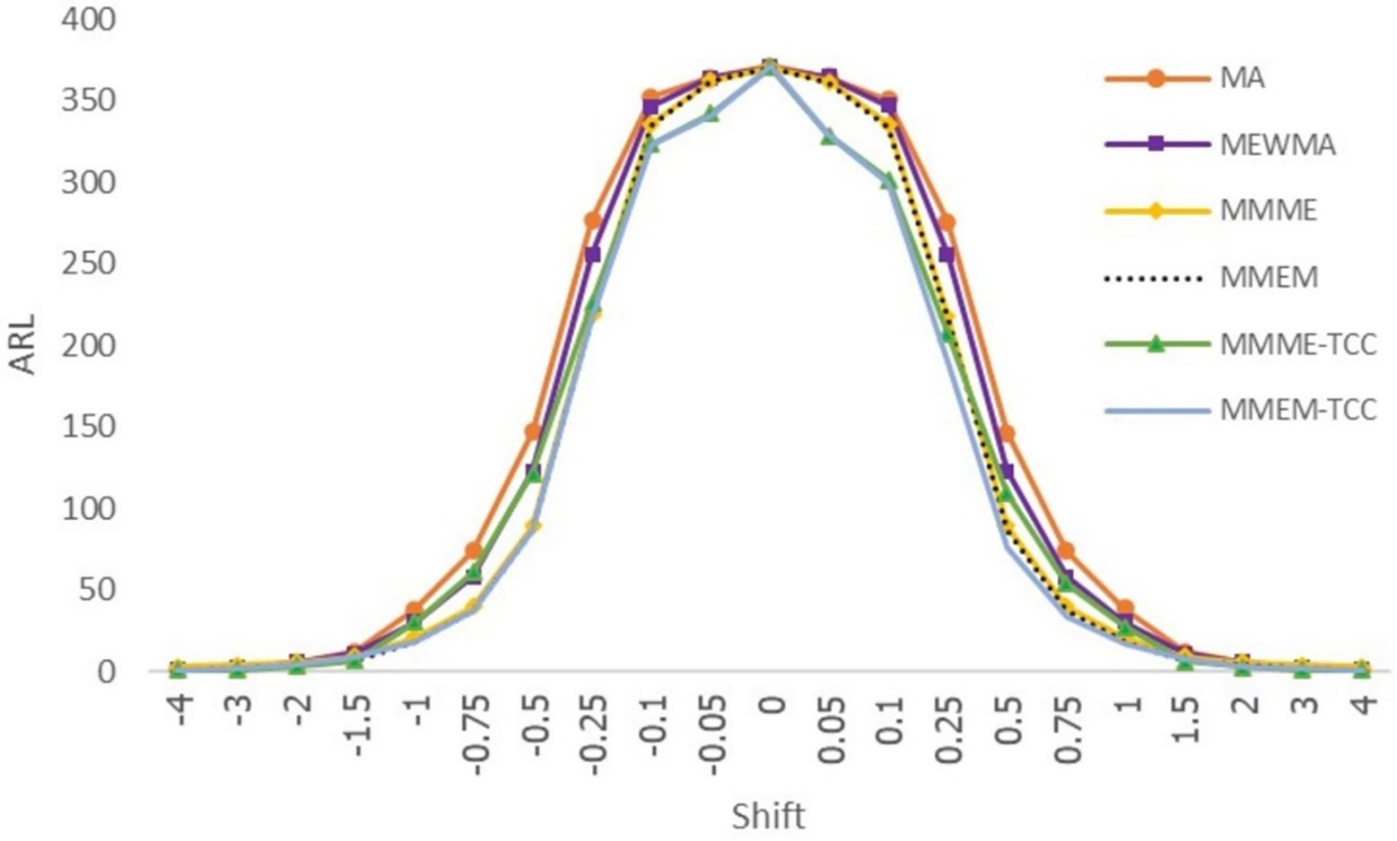
Displays of ARL curves of the MA, MEWMA, MMME, MMEM, MMME-TCC, and MMEM-TCC control charts for Laplace distribution.

When detecting shifts ±0.05, ±0.10, ±0.25, ±0.50, ±0.75, ±1.00, the proposed MMEM-TCC chart at *C*_*6*_ = *6*.*479* reduces the minimum ARL more than existing control charts when the process follows Laplace distribution. However, the MMME-TCC control chart outperforms when detecting shifts at ±1.50, ±2.00, ±3.00 and ±4.00. When the SDRL and MRL values were considered, the results were found to be correlated with ARL_1_, as shown in Table 2 and Fig 2.

**Fig 3:**
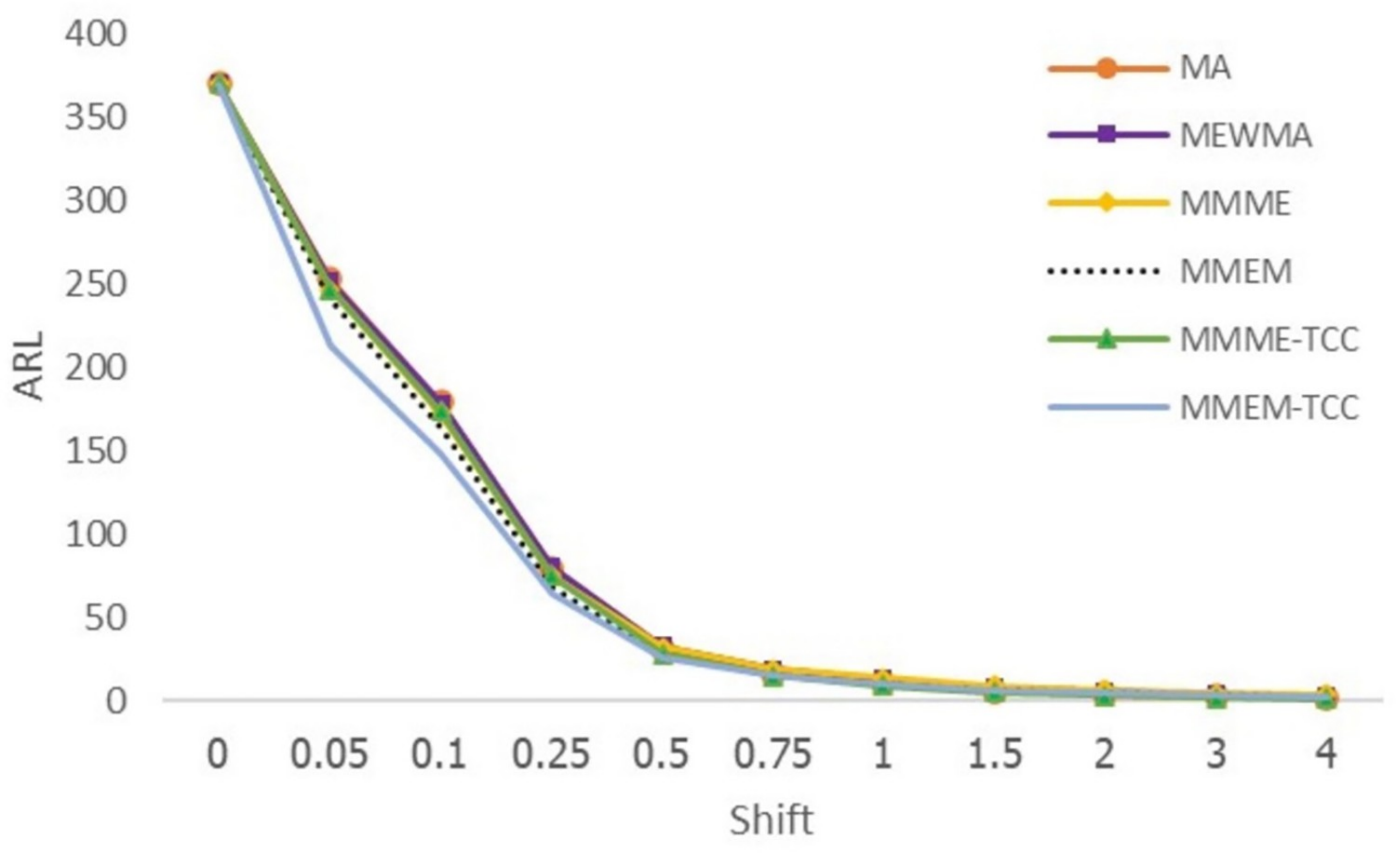
Displays of ARL curves of the MA, MEWMA, MMME, MMEM, MMME-TCC, and MMEM-TCC control charts for exponential distribution.

**Fig 4:**
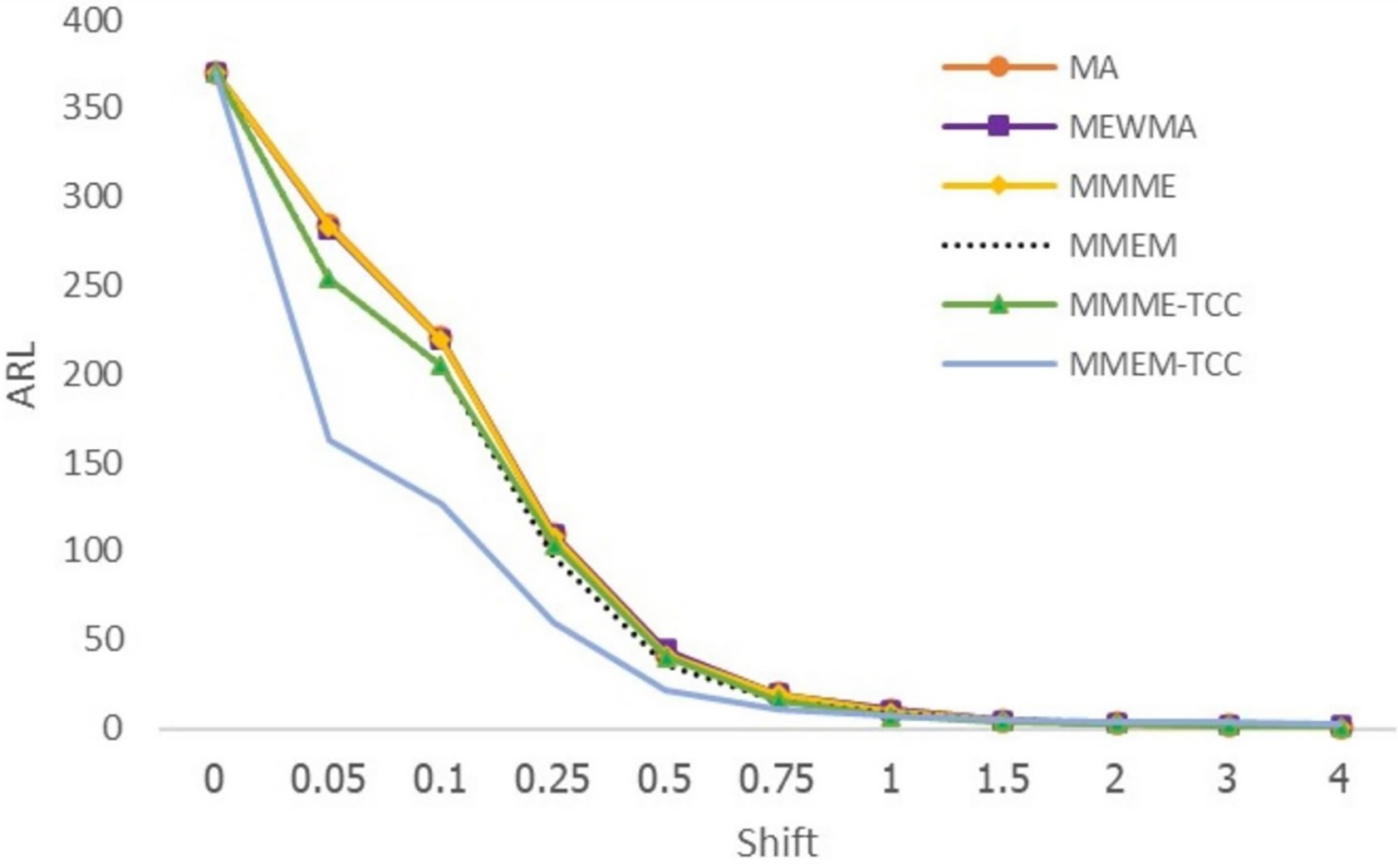
Displays of ARL curves of the MA, MEWMA, MMME, MMEM, MMME-TCC, and MMEM-TCC control charts for gamma distribution.

From Tables 3 and 4, Figs 3 and 4, when the process follows exponential and gamma distributions, the proposed MMEM-TCC chart detects small shifts more effectively when shifts are less than 1, while the MMME-TCC control chart is the most useful in detecting changes when shifts are equal or greater than 1. When the SDRL and MRL values were viewed, the results were found to be compatible with ARL_1_.

## Real data applications

We used the proposed control chart on two sets of real environmental data [32]. The first data set was PM 2.5 collected over a two-month period (November-December) in 2020 from the Chiang Mai Government Center Station, which had an exponential distribution. The second data set was from PM 10 between 12.00 and 13.00 pm. In 2021, data was collected for two months (November-December) from the Chiang Mai Government Center Station, which had gamma distribution. The Shapiro-Wilk goodness of fit test was used to clarify the distributions of two data sets as asymptotic exponential and gamma. The results indicate that the PM 2.5 data significantly fitted an exponential distribution (P-value = 0.2880 > 0.05) with a mean of 0.0396, while the PM 10 data already substantially fitted a gamma distribution (P-value = 0.8821 > 0.05) with the shape parameter 11.5527 and the scale parameter 3.2963, as shown in Table 5.

**Table 5:** Goodness-of-Fit test of real data.

Test distribution of data	PM 2.5	PM 10
Shapiro-Wilk test statistic	0.9764	0.9895
Estimated parameter(s)	rate = 0.0396	shape = 11.5527
		scale = 3.2963
P-value	0.2880*	0.8821*

**Fig 5:**
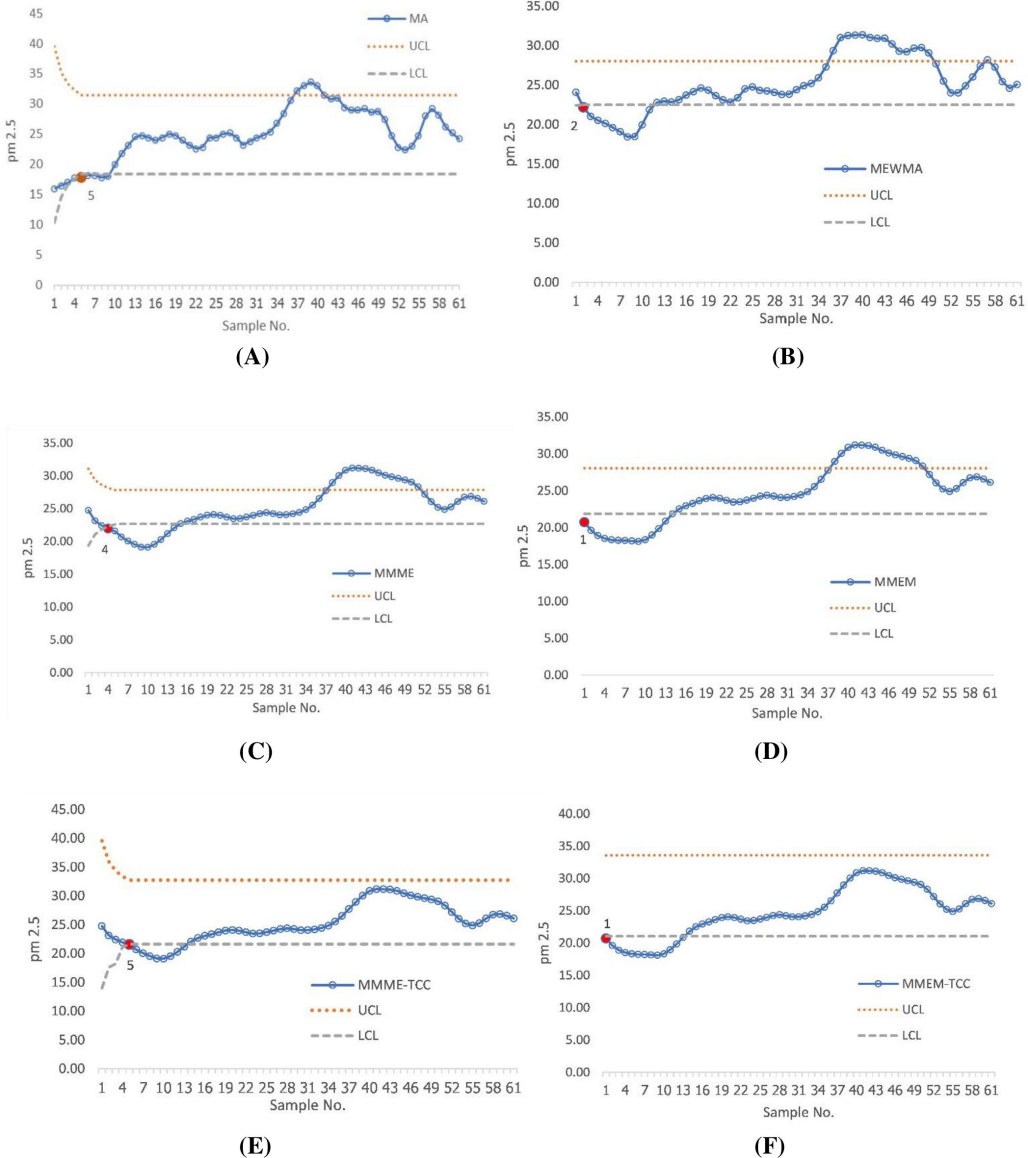
Displays of the PM 2.5 data set for the existing control chart (A) MA chart, (B) MEWMA chart, (C) MMME chart, (D) MMEM chart, (E) MMME-TCC, and (F) the proposed chart.

**Fig 6:**
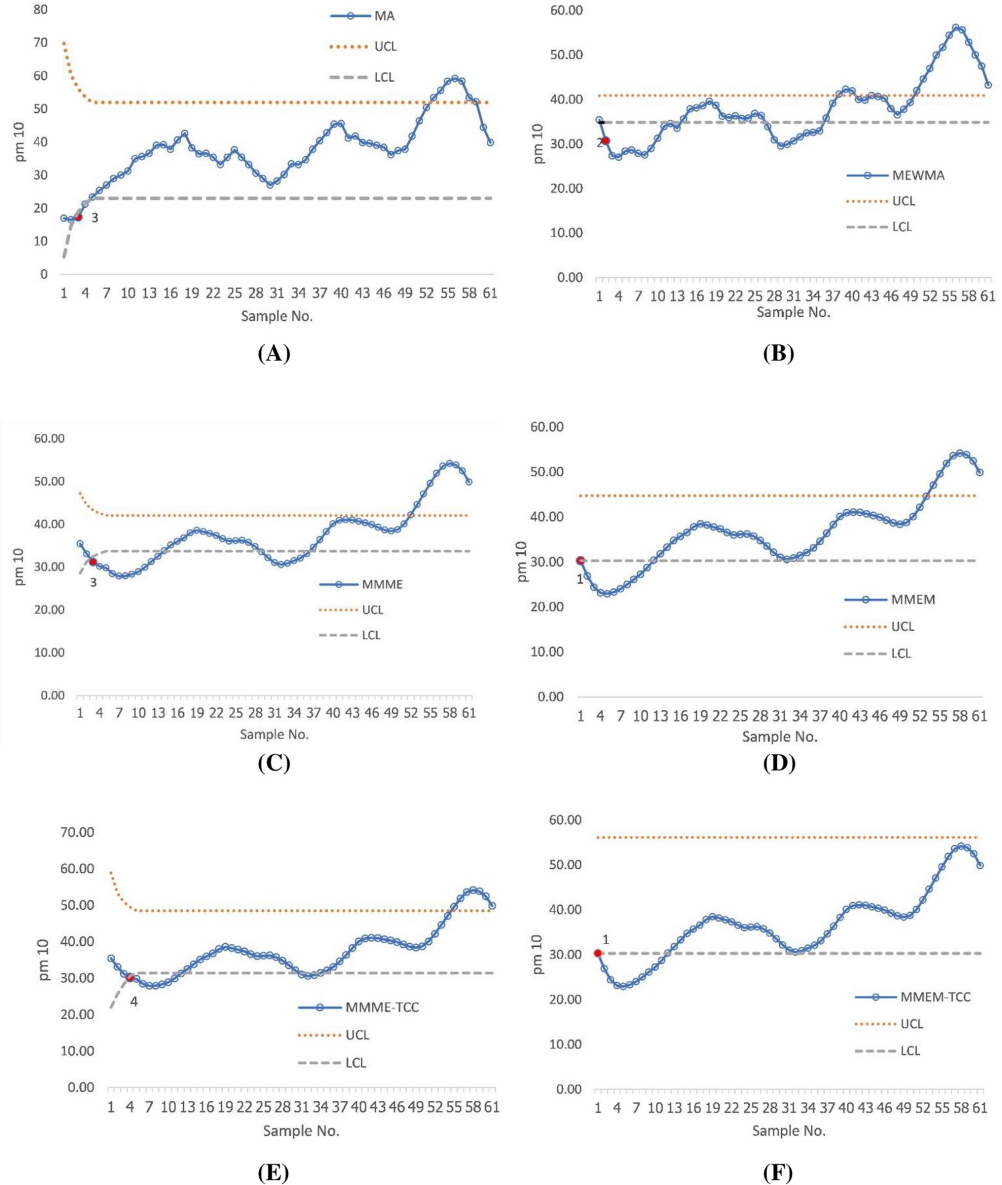
Displays of the PM 10 data set for the existing control chart (A) MA chart, (B) MEWMA chart, (C) MMME chart, (D) MMEM chart, (E) MMME-TCC, and (F) the proposed chart.

Fig 5 demonstrates the performance of all of the above-mentioned charts in the first data set. The results showed that both the proposed scheme and the MMEM chart detected the first out of control signal at the 1st sample. In the 2^nd^ sample, the MEWMA chart was performed to detect. In the 4^th^ sample, the MMME chart was performed to detect. At the 5^th^ sample, the MA and MMME-TCC charts were performed to detect. Furthermore, the control chart’s application to the second data set revealed that the proposed scheme and the MMEM chart were able to detect the change from the first time, the MEWMA control chart detected the second time, the MA and MMME charts detected the third time, and the MMME-TCC detected the fourth time, as shown in Fig 6.

In addition, we showed the results from the comparison of EARL values for period shift. Table 6 shows that when we look at total potential shift size, the EARL value of the proposed chart is always smaller than the other charts in all distributions. Although, if we evaluate a moderate to large shift in the procedure from Table 7, we can see that the EARL of the MMME-TCC chart is indeed slightly smaller than the other charts, excluding the Laplace distribution, where the proposed chart outperforms the others. Greatest importantly, we can see the proposed chart’s significant superiority over the other charts when we consider a small shift size from Table 8, so the EARL value of the proposed MMEM-TCC scheme becomes substantially smaller than the existing chats.

**Table 6:** Comparison of EARL values for the overall shift size of the charts’ performance.

Distribution	Shift size [0,4]					
	MA	MEWMA	MMME	MMEM	MMME-TCC	MMEM-TCC
Normal	15.70	13.97	14.56	12.30	13.18	**11.62**
Laplace	33.01	29.76	25.28	22.63	24.59	**20.59**
Exponential	12.29	14.21	15.21	11.92	11.14	**11.66**
Gamma	12.99	13.49	12.84	12.04	11.66	**9.70**

**Table 7:** Comparison of EARL values for a moderate to large shift size of the charts’ performance.

Distribution	Shift size [1,4]					
	MA	MEWMA	MMME	MMEM	MMME-TCC	MMEM-TCC
Normal	6.74	7.40	10.47	6.05	**5.49**	5.95
Laplace	19.71	18.89	17.98	12.36	13.01	**12.20**
Exponential	10.99	15.08	18.22	12.19	**9.27**	12.60
Gamma	7.69	8.61	7.48	8.49	**6.30**	10.59

**Table 8:** Comparison of EARL values for small shift size of the charts’ performance.

Distribution	Shift size [0,1]					
	MA	MEWMA	MMME	MMEM	MMME-TCC	MMEM-TCC
Normal	23.35	19.86	18.84	17.65	19.68	**16.55**
Laplace	48.30	42.07	33.70	32.60	36.94	**29.06**
Exponential	14.99	15.84	15.53	13.44	13.97	**12.79**
Gamma	18.32	18.51	17.99	15.77	16.34	**10.24**

## Discussion and conclusions

A MMEM-TCC control chart is proposed to effectively describe small and moderate shifts in process mean. The assessments and comparisons prove that the proposed MMEM-TCC chart outperforms the MA, MEWMA, MMME, MMEM, and MMME-TCC charts in terms of out-of-control average run length (*ARL*_*1*_) and EARL whereas, the MMME-TCC control chart can detect a large shift better than other charts. Additionally, we evaluated by comparing the ARL performance of the proposed chart to EWMA-TCC [24], MEC-TCC [25] and MDEWMA-TCC [33] under in-control average run length *ARL*_*0*_ = *370* and *λ* = *0*.*25*. The simulation results indicate that the proposed chart significantly outperform the EWMA-TCC for all dimensions of change under normal and asymmetric distributions, and that it exceeded the MEC-TCC for all dimensions of change under normal distribution. When compared to MDEWMA-TCC, the proposed chart was considered more efficient for minor shifts in the normal distribution. The results of the illustrative examples of the proposed chart for the two sets of data confirmed that the proposed chart was successful in detecting changes quickly in both data sets. Thus, the proposed chart displayed the sensitivity of monitoring schemes to detect small to moderate shifts. A mixed control chart combined with a nonparametric control chart provided an excellent choice for quality consultants and can be used in distribution-free statistics. This technique is applicable in other fields such as epidemiological data, health care, agricultural sectors, and so on. Future research might compare the data to other distributions or examine the comparison by adjusting the sample size, smoothing parameter, and additional parameter.

## Supporting information

S1 Data pm 2.5 and pm 10. (DOCX)
