# Supplementary material for: Performance of new nonparametric Tukey modified exponentially weighted moving average—Moving average control chart
Source: PLoS One. 2022 Sep 29;17(9):e0275260. doi: 10.1371/journal.pone.0275260 (PMC9522256; doi:10.1371/journal.pone.0275260)
Supplement: S1 Data — (DOCX) [file pone.0275260.s001.docx]

**Data PM 2.5**

| sample no. | PM 2.5 |
| --- | --- |
| 1 | 16 |
| 2 | 17 |
| 3 | 18 |
| 4 | 20 |
| 5 | 18 |
| 6 | 18 |
| 7 | 17 |
| 8 | 16 |
| 9 | 21 |
| 10 | 28 |
| 11 | 27 |
| 12 | 24 |
| 13 | 23 |
| 14 | 22 |
| 15 | 26 |
| 16 | 25 |
| 17 | 26 |
| 18 | 26 |
| 19 | 21 |
| 20 | 22 |
| 21 | 21 |
| 22 | 23 |
| 23 | 27 |
| 24 | 29 |
| 25 | 22 |
| 26 | 24 |
| 27 | 24 |
| 28 | 23 |
| 29 | 23 |
| 30 | 25 |
| 31 | 27 |
| 32 | 26 |
| 33 | 26 |
| 34 | 30 |
| 35 | 33 |
| 36 | 38 |
| 37 | 34 |
| 38 | 30 |
| 39 | 33 |
| 40 | 30 |
| 41 | 30 |
| 42 | 31 |
| 43 | 31 |
| 44 | 25 |
| 45 | 28 |
| 46 | 30 |
| 47 | 32 |
| 48 | 28 |
| 49 | 26 |
| 50 | 21 |
| 51 | 17 |
| 52 | 22 |
| 53 | 26 |
| 54 | 29 |
| 55 | 30 |
| 56 | 33 |
| 57 | 28 |
| 58 | 21 |
| 59 | 19 |
| 60 | 25 |
| 61 | 28 |

| **Data PM 10**  sample no. | PM10 |
| --- | --- |
| 1 | 17 |
| 2 | 16 |
| 3 | 19 |
| 4 | 33 |
| 5 | 32 |
| 6 | 27 |
| 7 | 24 |
| 8 | 29 |
| 9 | 38 |
| 10 | 38 |
| 11 | 46 |
| 12 | 27 |
| 13 | 34 |
| 14 | 50 |
| 15 | 39 |
| 16 | 39 |
| 17 | 41 |
| 18 | 44 |
| 19 | 28 |
| 20 | 30 |
| 21 | 40 |
| 22 | 35 |
| 23 | 33 |
| 24 | 39 |
| 25 | 41 |
| 26 | 29 |
| 27 | 24 |
| 28 | 20 |
| 29 | 31 |
| 30 | 31 |
| 31 | 35 |
| 32 | 34 |
| 33 | 36 |
| 34 | 30 |
| 35 | 38 |
| 36 | 51 |
| 37 | 47 |
| 38 | 48 |
| 39 | 43 |
| 40 | 39 |
| 41 | 29 |
| 42 | 50 |
| 43 | 38 |
| 44 | 42 |
| 45 | 36 |
| 46 | 26 |
| 47 | 39 |
| 48 | 44 |
| 49 | 44 |
| 50 | 56 |
| 51 | 49 |
| 52 | 59 |
| 53 | 59 |
| 54 | 55 |
| 55 | 70 |
| 56 | 53 |
| 57 | 55 |
| 58 | 34 |
| 59 | 49 |
| 60 | 31 |
| 61 | 30 |
